# Quality Appraisal of the Pharmacoeconomic Research Literature about Antivirals: A Comparison between Chinese Medicine and Non-Chinese Medicine

**DOI:** 10.1155/2021/5537435

**Published:** 2021-07-14

**Authors:** Junliang Zhang, Qian Bai, Ying Bian

**Affiliations:** State Key Laboratory of Quality Research in Chinese Medicine, Institute of Chinese Medical Sciences, University of Macau, Taipa, Macao, China

## Abstract

**Introduction:**

Antiviral activity is a main function of many types of traditional Chinese medicine (TCM), and they may contribute more in the process of certain viral epidemics. Therefore, based on the effectiveness and economy of TCM, we aimed to determine the situation of health economic studies about antivirals, especially the difference between TCM and non-TCM.

**Methods:**

A literature search of three databases was conducted with a time range of January 2000–December 2020, and terms related to health economics and TCM were used as key terms. QHES and CHEERS were used as quality assessment tools.

**Results:**

203 papers were included in our research. After evaluation using QHES and CHEERS, antiviral TCM obtained an overall score of 41.37 ± 4.46/99 in QHES, compared with 48.89 ± 7.25/99 (18.18% higher than TCM) of antiviral non-TCM.

**Conclusion:**

With a statistically significant difference, the overall quality of pharmacoeconomic research about antiviral non-Chinese medicine is better than that about antiviral Chinese medicine, which may have resulted from researchers' capacities or the absence of a more suitable standard for pharmacoeconomic research. It tells that the quality of pharmacoeconomic studies about TCM still warrants improvement.

## 1. Introduction

Viral infection has become one of the main causes of infectious diseases in humans. Reportedly, >10% of the world's population are infected with viruses like HIV and hepatitis C and B, all of which can cause severe diseases and even death during their progression [1]. In the course of fighting viruses, humanity has discovered thousands of natural products and invented medicines based on them. These medicines have saved many people's lives, resulting in a decrease of the mortality rate caused by virus infection [2, 3]. Meanwhile, the recent COVID-19 pandemic, which has posed tremendous threats to the global public health system, has made pharmacotherapy for COVID-19 a concern for many researchers and medical practitioners all over the world. Even though no specific medicine is recommended to prevent or treat COVID-19, it is still significant for us to know more about antiviral drugs, since drug therapy is the most efficient way to cure viral infection. According to some experts' estimations, the novel coronavirus (SARS-CoV-2) will coexist with human beings for a long time, seriously threatening our public health [4]. Therefore, as one of the most important weapons in the fight against pathogenic viruses, antiviral drugs, including traditional medicine, should receive attention and be valued, especially their safety, efficacy, and economic value.

Among all traditional medicines around the world, traditional Chinese medicine (TCM) has gained extensive attention because of its potential effectiveness and economic value since the COVID-19 outbreak in Wuhan, China. During the COVID-19 epidemic in China, several Chinese medicine prescriptions were promoted as routine prescriptions for the prevention and treatment of COVID-19 such as *qingfei paidu* decoction, *gancaoganjiang* decoction, and *sheganmahuang* decoction [5]. This marks that practitioners' recognition of TCM has risen to the level of clinical policy. Besides, as a main function of TCM, the antiviral effect in TCM has been researched in recent decades. In the process of discovering ways of treating COVID-19, researchers found a potential correlation between injections of Chinese medicine and some key targets through research methods like network pharmacology [6]. As for the activity of treating COVID-19, some classic proprietary Chinese medicines such as *lianhuaqingwen* capsules have been demonstrated to show antiviral and anti-inflammatory activity against novel coronavirus [7]. Cui et al. reviewed TCM for COVD-19 treatment and they summed up the clinical outcome, pathogenesis, and present application of TCM used to treat COVID-19 [8]. These could demonstrate the effectiveness of TCM in treating COVID-19 to some extent.

Considering that TCM could also function when used to treat viral infection, its safety, efficacy, and economy should be evaluated in clinical practice. However, studies evaluating the economics of TCM were unsatisfactory. Dujun reviewed the research development of pharmacoeconomic evaluation on TCM as of 2009 [9]. They found the literature on the economic evaluation of traditional Chinese medicine insufficient. Moreover, some problems existed in TCM pharmacoeconomic studies, including limited evaluation methods and less rigorous study design; therefore, the premarketing pharmacoeconomic evaluation on TCM remains a great necessity. These facts reveal the unsatisfactory quality of TCM's pharmacoeconomic evaluation.

Compared with other fields of disease, there are few kinds of antiviral drugs in the fight against viruses [10]. Both TCM and non-TCM antiviral medicine have taken obvious effect in tackling viral infections. However, there is no agreement as to whether TCM antiviral medicine is superior to non-TCM antiviral medicine from the perspectives of safety, efficacy, and economic value. The comparison between TCM and non-TCM antiviral medicine, especially from an economic perspective, might contribute to priority selection toward various treatment measurements for policymakers [11]. Together with problems in related pharmacoeconomic studies, it is important to conduct a rigorous quality appraisal in this field. Therefore, we aimed to evaluate the quality of current pharmacoeconomic analysis on antiviral drugs while also comparing the quality between TCM and non-TCM antiviral medicines using main health economic evaluation tools.

## 2. Methods

### 2.1. Data Source

The literature to be appraised consisted of the Web of Science, PubMed, and China National Knowledge Infrastructure (CNKI). The former two databases encompass the bulk of studies in biomedicine and natural and social science in different countries. CNKI is China's largest knowledge resource sharing grid platform, covering a large number of studies in every field. Therefore, pharmacoeconomic studies concerning antivirals were retrieved from the abovementioned databases.

### 2.2. Search Strategy

Based on the classification of health economic analysis and the research topic of antiviral drugs, we determined final research strategy as “cost-effectiveness” OR “cost-minimization” OR “budget impact analysis” OR “cost-benefit” OR “cost-utility” OR “pharmacoeconomic” OR “health economic” AND “(traditional) Chinese medicine” OR “antiviral/anti-virus.” In the same way, we search these terms in Chinese in CNKI to find the relevant literature written in Chinese. Publishing date of literature was confined from January 2000 to December 2020.

### 2.3. Inclusion and Exclusion Criteria

Literature with the following characteristics is included: (1) introducing original pharmacoeconomic research about antiviral chemicals or Chinese medicine with antiviral effect; (2) original health economic research on strategies that only use antiviral drugs to treat or prevent disease instead of vaccines, medical instruments, public policies, etc.; (3) papers that meet the above criteria with accessibility to the full text.

The literature with the following features would be excluded: (1) theses or dissertations that combine pharmacoeconomic studies with other content; (2) studies about pharmacoeconomic evaluation of combinations containing both Chinese and Western medicine, or both antiviral drugs and non-antiviral drugs; (3) duplicate versions that were repeatedly published or published in another language; (4) literature of news, meetings, letters, and reviews, especially those papers as an introduction to some completed pharmacoeconomic studies or methodology that were not empirical studies.

### 2.4. Quality Assessment

Quality of Health Economic Studies (QHES) is a validated instrument for the critical appraisal in health economic evaluation. QHES provides a checklist with 16 items, each of which is connected to specific points that also represent their weights in the whole quality assessment. By using this checklist to quantitatively evaluate the literature, each included item will get a total score that could be used in an intuitive comparison of literature quality.

In addition, Consolidated Health Economic Evaluation Reporting Standards (CHEERS) is designed to standardize and improve the reporting quality of health economic evaluations. With 24 items to be measured, CHEERS can efficiently avoid the influence of subjectivity to a certain extent, by defining the evaluation degree of the items. At the same time, CHEERS gives us a grading standard, making it complementary to QHES in actual practice. To calculate the score of each paper, we assigned a weight of “1” to papers marked “fully reported,” “0.5” to those marked “partially reported,” and “0” to those marked “unreported (if applicable).” We used QHES and CHEERS as our evaluation tools for the included literature.

In terms of the differences between the two tools above, it should be noted that the items in both checklists overlap in part.  Table 1 shows the content of the items in the checklists for both tools in detail. Besides, since the weights of each level in CHEERS assessment were not designed when CHEERS was invented, unlike QHES, the scores based on the CHEERS checklist are solely for reference and the scoring results between QHES and CHEERS are not comparable [12, 13].

### 2.5. Statistics

SPSS Statistics 21 was used to calculate the weighted mean differences (MD), the value of OR, and the 95% confidence interval in data analysis. The Chi-square test for discrete data and *t*-test for continuous data were applied in our study and *P* < 0.05 was regarded as being statistically significant, which was marked in Table 1.

## 3. Results

**Figure 1:**
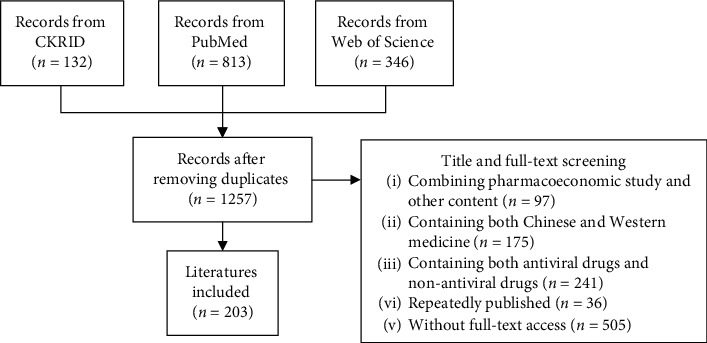
Flow diagram of the search process.

Overall, 1,291 studies in the literature were extracted from the three databases, leaving 1,257 studies after removing duplicates. After title and full-text screening, 203 studies remained.  Figure 1 is a flow diagram of the detailed search process.

Basically, Table 2 shows the situation of the included literature, which contains the classification based on several items within it. From the perspective of written language, 75.86% of all included papers were in English, while the other 24.14% were in Chinese. Generally, the pharmacoeconomic papers about antivirals written in Chinese were still fewer than those in English. Regarding the published year, there was a growing trend of antiviral publications every five years. It seems that more practitioners paid more attention to the field of pharmacoeconomic evaluation. In terms of the type of drugs, nucleoside analogs including entecavir, lamivudine, and adefovir seized the largest proportion of all literature. This was consistent with the actual use of drugs in clinical practice [14]. Hereafter, the model analysis was not widely used because 94.58% of all papers did not describe their model analysis. As for the analysis method, cost-effectiveness analysis (CEA) was undoubtedly the most common method, while budget impact analysis (BIA) was second, accounting for 8.37%; few papers chose CBA or CUA as their analysis method. Sixty-five point zero two percent of all included literature were retrospective while 34.98% were prospective. Seventy-eight point eight two percent of investigated papers were not funded, and SPSS was the most popular analysis software among the literature at 46.80%. Stata, SAS, and Microsoft Excel were also mentioned as having been used.

Table 3 illustrates the scoring result using the QHES checklist. The average scores and standard deviations of 15 items were calculated. In general, Items 12–14 and 16 showed an extremely low result score. Considering the reference range, Item 13, which represented the statement situation of the economic model, assumption, and limitation, showed the worst performance among all items in the QHES checklist. Item 4 (reference range: 0–1) refers to subgroup analysis and relevant prespecification, while none of the included literature covered subgroup analysis, and so Table 3 does not display Item 4.

The results shown in Table 4 were in accordance with the grading system of the CHEERS checklist. Twenty-three items were taken into consideration, and Items 1 (98.52% fully reported), 3 (97.04% fully reported), 5 (99.51% fully reported), and 10 (96.06% fully reported) showed excellent reporting results. Some items, like Item 9 that was 94.09% unreported, did not show an ideal result of reporting. Item 12 (measurement and valuation of preference-based outcomes) was not presented because none of the included studies were related to preference-based outcomes.

Since the evaluation result of QHES is quantitative, a comparison between Chinese medicine and non-Chinese medicine could be shown in an intuitive way as given in Table 5.

From all the data above, we could know the differences between antiviral TCM and non-TCM in a statistical manner. The scores for Items 7, 12, and 13 of non-TCM antiviral studies were significantly higher than those of the TCM antiviral studies with *P*-values <0.05. The scores of TCM antiviral studies were only higher than those of non-TCM studies for Items 1 and 9. Higher scores occurred in non-TCM antiviral literature for the remaining items.

## 4. Discussion

With the application of QHES and CHEERS, this study evaluated the reporting quality of pharmacoeconomic studies concerning antiviral medicine and the differences between TCM and non-TCM antiviral medicine. Findings identified that there was a gap in the actual situation of included pharmacoeconomic studies and the ideal quality level. Besides, the score of TCM antiviral medicine evaluations turned out to be slightly lower than those of non-TCM evaluations in most QHES items.

Obviously, the quality of literature about antiviral Chinese medicine was not high. It is believed that the pharmacoeconomic studies about TCM were generally of a low level. In 2008, Li et al. reported that they had analyzed the pertinent literature from several aspects such as author affiliation, research method, research perspective, research object, and research duration and found that many problems still remained in Chinese pharmacoeconomic evaluation studies [14]. Considering the abovementioned circumstance, Wang et al. established guidelines for the pharmacoeconomic evaluation of proprietary Chinese medicine to make the evaluation process and results both scientific and fair [15]. It was convincing that subsequent pharmacoeconomic researchers could access this guideline and follow it consciously [16, 17].

According to the results, the *P*-values of Items 7, 12, and 13 and the total score in Table 5 were <0.05, which meant the scores of literature quality were significantly different in these aspects. Fourteen of the 16 items showed that the scores of antiviral non-TCM were higher than those of TCM. Item 7 refers to whether the data abstraction methodology was stated, Item 12 refers to the clarity and transparency with which the economic model, study methods, etc. were displayed, while Item 13 is about the statement of the choice of economic model, main assumption, and limitation. These imply that there is still a way to go for health economic researchers focusing on TCM, especially in the rigor of matters about the economic models and the awareness of clarifying the methodology in their empirical research.

Meanwhile, there were several potential reasons that resulted in such a situation of literature quality. Most pharmacoeconomic studies were conducted by medical workers in hospitals, especially pharmacists [18]. It was mentioned that the pharmacist conducting the pharmacoeconomic evaluation is most likely to obtain effective and useful results by following the described analysis steps [19]. However, the need remains to improve the quality of studies conducted by pharmacists. For medical workers in China, their pharmacoeconomic evaluations probably arose from their hopes of title promotion. Since practitioners in medical institutions can be accessible to many patients who need to take drugs periodically, it is easier for them to conduct health economic evaluations, like comparisons of the cost-effectiveness ratio of two commonly used drugs in clinics [20, 21]. Therefore, most of their original motivation was not the economy of health insurance funding or affordability for patients, but their personal benefit. This is an influential factor in the literature quality.

These evaluations are of great significance because they could provide evidence for doctors and patients when choosing which drugs to use. It could also save funds for medical insurance and the government could redistribute the money saved to healthcare workers, thus increasing their incomes. Farid et al. reported that, in developing countries such as Egypt, the growing population was a threat to the allocation of the domestic health system. Medical resource scarcity exists in almost all countries and regions and if the quality of health economic evaluations could be improved, officials in national medical departments can make better decisions [22]. Besides, Acharya introduced the urgent need of pharmacoeconomic studies in Nepal [23]. For nations with little funding to develop their medical industry, it is hard to start from zero, not to mention improving studies' quality. Therefore, it is also high time for pharmacoeconomics researchers to notice normative standards in health economic studies.

Regarding the limitations of this study, there may be selection bias for included papers. Language and regional barriers are restrictions for authors to search for more papers that meet the inclusion criteria. It could be difficult for many researchers at home to read publications in foreign journals, especially those published in a foreign language [24, 25]. For example, if a Japanese scholar wants to look for some information about Chinese medicine, they might find it difficult to obtain access to certain Chinese databases that include studies about Chinese medicine because the scholar's institute has not paid for the databases or for other reasons. In this research, we focused on literature in English and Chinese, while papers written in other languages were not considered, which might cause selection bias.

During the appraisal period, some disadvantages of the QHES and CHEERS checklists appeared to be unscientific to some extent [26] because many studies provided more information about methodology, explanations about bias, and so on in detail. Besides, the subjective score would bring injustice to the scoring results. A new checklist of an evaluation tool that is specific for pharmacoeconomic studies is needed and it may solve these problems in later practice [27].

## 5. Conclusions

Against the background of few antivirals found by humans, the significance of antiviral drugs, including TCM and non-TCM, was highlighted. A quality appraisal in pharmacoeconomics was urgently needed to reveal the shortcomings of certain modern evaluations in the field of fighting against virus infection. TCM is proved to be safe, effective, and economic when used to treat virus infection, and this led to the original purpose of our research. In our research, the overall level of literature's quality about antiviral TCM is still low at 41.37 ± 4.46 in the QHES scoring compared to 48.89 ± 7.25 of antiviral non-TCM. This may be due to the executors of pharmacoeconomic studies, most of whom were pharmacists. Thus, space remains for improving their educational background and clarifying their inspiration for conducting economic research to make their studies more purposeful and of higher quality. One more thing that warrants attention is that a new evaluation tool that is better suited to pharmacoeconomics needs to be invented to meet modern scientific research demands.

## Figures and Tables

**Table 1:** Comparison of item content between QHES and CHEERS.

Item content	QHES	CHEERS
Title and abstract		√
Epidemiological information		√
Target population		√
Time horizon	√	√
Objectives	√	√
Assumptions		√
Perspective	√	√
Comparators		√
Choice of model	√	√
Discount	√	√
Data processing and analysis methods		√
Data source	√	√
Definition and calculation of costs	√	√
Definition and calculation of outcomes	√	
Incremental costs and outcomes	√	√
Sensitivity analysis	√	√
Heterogeneity analysis		√
Results	√	√
Funding sources or stakeholder group	√	√
Discussion of limitations	√	√

**Table 2:** Description of the included literature's information.

Item		Number (%)
*Language*	English	154 (75.86)
	Chinese	49 (24.14)

*Publication year*	2000–2004	10 (4.92)
	2005–2009	32 (15.76)
	2010–2014	74 (36.45)
	2015–2020	87 (42.87)

*Type of drug*	Nucleoside analog	90 (44.33)
	Chinese medicine	53 (26.11)
	Interferon	38 (18.72)
	Others	22 (10.84)

*Model analysis*	Described	11 (5.42)
	Not described	192 (94.58)

*Analyzing method*	CEA	148 (72.91)
	CBA	6 (2.96)
	CUA	2 (0.99)
	BIA	17 (8.37)
	Others	30 (14.77)

*Design method*	Retrospective study	132 (65.02)
	Prospective study	71 (34.98)

*Fund*	Yes	43 (21.18)
	No	160 (78.82)

*Software used*	SPSS	95 (46.80)
	Stata	9 (4.43)
	Others	99 (48.77)

**Table 3:** QHES scoring result of methodological quality evaluation.

Item	Score	Reference range
Item 1	3.91 ± 2.26	0–7
Item 2	1.66 ± 0.21	0–4
Item 3	5.46 ± 0.93	0–8
Item 5	4.69 ± 2.17	0–9
Item 6	3.31 ± 2.62	0–6
Item 7	1.64 ± 0.73	0–5
Item 8	5.48 ± 1.65	0–7
Item 9	5.54 ± 0.54	0–8
Item 10	4.34 ± 1.95	0–6
Item 11	4.07 ± 1.19	0–7
Item 12	0.78 ± 2.16	0–8
Item 13	0.21 ± 0.38	0–7
Item 14	0.64 ± 1.43	0–6
Item 15	3.73 ± 1.05	0–8
Item 16	0.16 ± 0.32	0–3
Total (except Item 4)	45.31 ± 11.47	0–99

**Table 4:** Reporting quality results based on the CHEERS checklist.

Item	Fully reported		Partially reported		Unreported	
	*n* (%)	95% CI	*n* (%)	95% CI	*n* (%)	95% CI
Item 1	200 (98.52)	(0.946, 0.992)	0 (0.00)	(0, 0.032)	3 (1.48)	(0.007, 0.021)
Item 2	17 (8.37)	(0.072, 0.093)	179 (88.18)	(0.864, 0.909)	7 (3.45)	(0.031, 0.037)
Item 3	197 (97.04)	(0.962, 0.983)	6 (2.96)	(0.013, 0.416)	0 (0.00)	(0, 0.032)
Item 4	134 (66.00)	(0.613, 0.714)	69 (33.99)	(0.245, 0.465)	0 (0.00)	(0, 0.032)
Item 5	202 (99.51)	(0.947, 0.999)	0 (0.00)	(0, 0.032)	1 (0.49)	(0.001, 0.048)
Item 6	24 (11.82)	(0.103, 0.154)	162 (79.80)	(0.612, 0.863)	27 (13.30)	(0.750, 0.231)
Item 7	43 (21.18)	(0.190, 0.312)	160 (78.82)	(0.645, 0.797)	0 (0.00)	(0, 0.032)
Item 8	3 (1.48)	(0.007, 0.021)	200 (98.52)	(0.946, 0.992)	0 (0.00)	(0, 0.032)
Item 9	9 (4.43)	(0.334, 0.475)	3 (1.48)	(0.007, 0.021)	191 (94.09)	(0.915, 0.968)
Item 10	195 (96.06)	(0.951, 0.973)	0 (0.00)	(0, 0.032)	8 (3.94)	(0.132, 0.429)
Item 11	42 (20.69)	(0.193, 0.211)	160 (78.82)	(0.758, 0.810)	1 (0.49)	(0.001, 0.048)
Item 13	174 (85.71)	(0.856, 0.861)	20 (9.85)	(0.083, 0.122)	9 (44.33)	(0.413, 0.476)
Item 14	65 (32.02)	(0.946, 0.992)	133 (65.52)	(0.589, 0.682)	5 (2.46)	(0.023, 0.027)
Item 15	11 (5.42)	(0.050, 0.076)	9 (4.43)	(0.038, 0.047)	183 (90.15)	(0.876, 0.946)
Item 16	11 (5.42)	(0.050, 0.076)	10 (4.93)	(0.043, 0.058)	182 (89.66)	(0.879, 0.921)
Item 17	131 (64.53)	(0.608, 0.661)	36 (17.73)	(0.160, 0.183)	36 (17.73)	(0.134, 0.193)
Item 18	8 (3.94)	(0.016, 0.044)	195 (96.06)	(0.943, 0.988)	0 (0.00)	(0, 0.032)
Item 19	102 (50.25)	(0.475, 0.539)	44 (21.47)	(0.201, 0.232)	57 (28.02)	(0.226, 0.329)
Item 20	21 (10.34)	(0.094, 0.116)	94 (46.31)	(0.392, 0.514)	88 (43.35)	(0.346, 0.471)
Item 21	146 (71.32)	(0.702, 0.731)	0 (0.00)	(0, 0.032)	57 (28.68)	(0.235, 0.313)
Item 22	19 (9.36)	(0.082, 0.117)	184 (90.64)	(0.876, 0.931)	0 (0.00)	(0, 0.032)
Item 23	24 (11.82)	(0.099, 0.136)	0 (0.00)	(0, 0.032)	179 (88.18)	(0.841, 0.932)
Item 24	2 (0.98)	(0.004, 0.015)	4 (1.97)	(0.011, 0.029)	197 (97.05)	(0.962, 0.983)
Total *X* ± SD (range)	13.44 ± 0.73 (9–16.5)					

**Table 5:** Comparison of QHES scores of Chinese medicine and non-Chinese medicine.

	Antiviral Chinese medicine	Antiviral non-Chinese medicine	MD	*P* value
Item 1	4.02 ± 3.48	3.85 ± 2.12	0.32 (0.12, 0.51)	0.43
Item 2	1.52 ± 0.31	2.14 ± 0.35	−0.65 (−0.86, −0.43)	0.12
Item 3	4.49 ± 0.70	5.52 ± 0.56	−1.04 (−1.36, −0.68)	0.65
Item 5	4.23 ± 3.13	5.21 ± 0.84	−1.17 (−1.76, −0.63)	0.44
Item 6	3.18 ± 4.91	3.54 ± 1.56	−0.46 (−0.63, −0.42)	0.62
Item 7	0.79 ± 1.22	1.93 ± 0.71	−1.16 (−1.32, −1.08)	0.01^*∗*^
Item 8	5.25 ± 1.37	5.85 ± 2.11	−0.61 (−0.84, −0.47)	0.56
Item 9	5.61 ± 0.63	5.52 ± 0.45	0.15 (0.12, 0.17)	0.83
Item 10	4.25 ± 1.99	4.51 ± 1.27	−0.39 (−0.42, −0.33)	0.59
Item 11	3.73 ± 0.54	4.18 ± 1.89	−0.47 (−0.81, −0.10)	0.43
Item 12	0.06 ± 5.13	1.18 ± 4.64	−1.02 (−1.64, −0.56)	≤0.01^*∗*^
Item 13	0.16 ± 2.46	0.77 ± 0.41	−0.59 (−0.67, −0.54)	0.03^*∗*^
Item 14	0.43 ± 0.36	0.74 ± 2.03	−0.36 (−0.44, −0.32)	0.16
Item 15	3.21 ± 0.12	3.84 ± 1.98	−0.72 (−0.81, −0.66)	0.51
Item 16	0.16 ± 1.03	0.16 ± 1.34	−0.01 (−0.37, −0.29)	0.98
Total	41.37 ± 4.46	48.89 ± 7.25	−7.46 (−7.71, −7.02)	0.03^*∗*^

^*∗*^ *P* < 0.05.

## Data Availability

The data supporting the findings of this study are included within the article.
